# BLACK MEN’S HEALTH AND AGING ACROSS THE LIFE COURSE

**DOI:** 10.1093/geroni/igae098.1595

**Published:** 2024-12-31

**Authors:** Roland Thorpe, Carl V Hill

**Affiliations:** Chair; Co-Chair

## Abstract

Much of the research on Black men tend not to focus on concepts that have been found to be important determinants of health for Black men. Nor, do they seek to identify sources of resilience that may be protective of health. This symposium contains a collection of papers that discuss some key social determinants of health (SDOH) that can provide insights to advance our understanding of Black men’s health and aging. Bruce and colleagues examined the association between religious practices and/or beliefs, socioeconomic status, and cognitive status among Black men in the 2016 Health and Retirement Study (HRS). Findings indicate private prayer was inversely related to any cognitive impairment among Black men with more than 12 years of education. Nwaskasi and colleagues examined the effect of active coping and grit on subjective cognitive decline (SCD) among Black men and found that active coping and grit were significant predictors of SCD while controlling for demographic variables. Esiaka and colleagues investigated the relationship between food insecurity and cancer screening behaviors among Black men dwelling in food deserts. Understanding the link between food insecurity and cancer screening behavior provides crucial insights for tailoring targeted interventions for Black men. Thomas Tobin and colleagues investigated the link between psychosocial resilience and allostatic load in Black men in the Nashville Stress and Health Study. Results from latent class analyses suggest different resource combinations produce distinct patterns of resilience among Black men. These presentations will bolster our knowledge on SDOH among Black men across the life course.

